# Temporal migration patterns and mating tactics influence size-assortative mating in *Rana temporaria*

**DOI:** 10.1093/beheco/arx188

**Published:** 2018-01-10

**Authors:** Carolin Dittrich, Ariel Rodríguez, Ori Segev, Sanja Drakulić, Heike Feldhaar, Miguel Vences, Mark-Oliver Rödel

**Affiliations:** 1 Museum für Naturkunde, Leibniz Institute for Evolution and Biodiversity Science, Berlin, Germany; 2 TU Braunschweig, Zoologisches Institut, Evolutionsbiologie, Braunschweig, Germany; 3 University of Veterinary Medicine Hannover, Institute of Zoology, Hannover, Germany; 4 University of Haifa, Faculty of Science, Institute of Evolution, Community Ecology Lab, Haifa, Israel; 5 University of Bayreuth, Animal Population Ecology, Bayreuth, Germany; 6 Berlin-Brandenburg Institute of Advanced Biodiversity Research (BBIB), Berlin, Germany

**Keywords:** amphibia, evolution, male–male competition, reproductive strategy, assortment by chance

## Abstract

Assortative mating is a common pattern in sexually reproducing species, but the mechanisms leading to assortment remain poorly understood. By using the European common frog (*Rana temporaria*) as a model, we aim to understand the mechanisms leading to size-assortative mating in amphibians. With data from natural populations collected over several years, we first show a consistent pattern of size-assortative mating across our 2 study populations. We subsequently ask if assortative mating may be explained by mate availability due to temporal segregation of migrating individuals with specific sizes. With additional experiments, we finally assess whether size-assortative mating is adaptive, i.e. influenced by mating competition among males, or by reduced fertilization in size-mismatched pairs. We find that size-assortative mating is in accordance with differences in mate availability during migration, where larger individuals of both sexes reach breeding ponds earlier than smaller individuals. We observe an indiscriminate mate choice behavior of small males and an advantage of larger males pairing with females during scramble competition. The tactic of small males, to be faster and less discriminative than large males, may increase their chances to get access to females. Experimental tests indicate that the fertilization success is not affected by size assortment. However, since female fecundity is highly correlated with body size, males preferring larger females should maximize their number of offspring. Therefore, we conclude that in this frog species mate choice is more complex than formerly believed.

## Teaser

Birds of a feather flock together. This is also true for the European common frog where mating of similar sized individuals is more likely. But, how they assort is a rather complex mechanism. Aside from mate choice behavior and male–male competition, size dependent temporal migration patterns cause assortative mating. Larger males and females reach breeding ponds earlier in the season and are thus more likely to meet.

## INTRODUCTION

Reproductive success is the most important aspect of individual fitness. Consequently, various mating systems, strategies, and tactics have evolved, and they may vary between and within species (Gross 1996; Shuster and Wade 2003). Random mating would mean that all individuals of a given population would mate with the same probabilities, but due to natural and sexual selection, physical constraints, and stratification of populations, nonrandom mating is the rule in taxa with sexual reproduction (Crespi 1989; Otronen 1993; Arnqvist et al. 1996; Harari et al. 1999; Bearhop et al. 2005; Taborsky et al. 2009). One common pattern of nonrandom mating is assortative mating, defined by the correlation of traits (phenotypic or genotypic) across mated pairs. Although the strength of assortment differs between taxa and traits, the direction of assortment is usually positive, i.e. individuals with similar traits are more likely to mate (Thiessen and Gregg 1980; Crespi 1989; Acord et al. 2013; Jiang et al. 2013). Negative assortment occurs if offspring may have advantages from trait dissimilarity of their parents, e.g. assortment to maximize diversity of major histocompatibility complex alleles (Meyer and Thomson 2001; Mays and Hill 2004), or advantages of heterozygotes (Hedrick et al. 2016). Assortment can also be incidental, due to spatial or temporal segregation (Jiang et al. 2013). Examples for such incidents causing assortative mating include spatial and/or temporal separation in birds (Bearhop et al. 2005), temporal segregation of *Drosophila* strains (Tauber et al. 2003), or differences in flowering periods in plants (Devaux and Lande 2008; Weis et al. 2014).

In anuran amphibians (frogs and toads), size-assortative mating is frequently observed, but the underlying causes have rarely been elucidated (Arak 1983; Halliday 1983; Howard and Kluge 1985; Sullivan et al. 1995). Mostly, size assortment is associated with male mate choice; when males compete directly over females, the access to females is limited, and the fertility of females is size dependent (Krupa 1995). A limited access to females leads to high variation in male mating success (Jones et al. 2002). Therefore, competition among males for females is common and considerably high in explosive or lek-breeding species (Wells 1977; Arak 1983; Bradbury and Gibson 1983). This competition can be expressed as direct combat between males, dominance of specific males, territoriality, or other tactics—e.g. satellite males—to gain access to females (Wells 1977; Shine 1979; Arak 1983; Tsuji and Matsui 2002). These mating tactics are often not fixed and the behavior of a nonpaired individual is status and context dependent and may thus change over its lifetime (Dominey 1984; Lucas et al. 1996; Bowcock et al. 2013). Fertility of anuran females is usually positively correlated with female body size (Wells 2007; Nali et al. 2014) and, therefore, males should prefer to mate with larger females to increase their reproductive fitness.

The first and most obvious scenario leading to size-assortative mating relies on competitive advantages of large males securing mating with the preferred large females (Berven 1981; Howard and Kluge 1985), e.g. due to their stronger grip in amplexus and better combat performance. Thus, pairs of large individuals are formed while small “left-over” females would mate with similarly small males. A second mechanism that could lead to assortative mating derives from the fact that reproductive success does not merely depend on the total number of eggs produced by a female but rather on the number of “fertilized” eggs sired by a male. In various explosive breeding anurans multiple paternity has been observed (Laurila and Seppä 1998; Lodé and Lesbarrères 2004; Vieites et al. 2004), which can occur through other males fertilizing those eggs that were left unfertilized by the amplecting male. This suggests that a substantial proportion of eggs are not immediately fertilized by the amplecting male. In particular, the distance between female to male cloaca may influence fertilization success, and thus fitness of mates in species with external fertilization such as most anurans (Davies and Halliday 1977; Robertson 1990). A third proximate factor that could lead to size assortment is the temporal sorting of differently sized individuals, where individuals of similar size arrive at similar times at the breeding sites (Howard and Kluge 1985; Ryser 1989; Elmberg 1990; Lodé et al. 2005). This could be due to physiological reasons (Morbey and Ydenberg 2001), e.g. larger individuals can store more energy reserves, have higher migration abilities, are less prone to desiccation, and could therefore start migration earlier under less favorable weather conditions (Elmberg 1991; Kovar et al. 2009). Furthermore, individuals hibernate in different overwintering sites and distances to ponds vary (Pasanen and Sorjonen 1994).

The European common frog, *Rana temporaria* Linnaeus, 1758, is a widespread Palearctic species and occurs in a variety of different habitats. Common frogs are explosive breeders; individuals aggregate in large numbers at the breeding sites for approximately 2 weeks in early spring (Gollmann et al. 2014). Usually, the operational sex ratio (OSR) at the breeding site is male-biased (Elmberg 1990; Vojar et al. 2015), which leads to male–male competition. Males show different mating tactics that seem to be size and frequency dependent. Small males can be seen searching/waiting for females at the edge of the breeding pond, while larger males seem to aggregate within the breeding pond, participating in scramble competition (Arak 1983). These larger males are more often successful in female takeover attempts than the smaller ones (Savage 1961). Therefore, it should be beneficial and cost effective for smaller males to be less picky in choosing a mate, also known as the concept of prudent choice (Härdling and Kokko 2005). If they are faster in grabbing a female, the chance to keep a female until spawning is increasing. We therefore hypothesize that, based on a combination of male mate choice, male–male competition and an evolutionary advantage of maximized fertilization success by size-matched pairs, sexual selection in *R. temporaria* might result in size assortment of mates. Additionally, incidental assortment due to migration patterns could favor assortment. Here, we use field data from 2 *R. temporaria* populations and experiments, to examine the mechanisms leading to pair-formation in populations of *R. temporaria.* We differentiated between mechanisms leading to size assortment during the migration period to the breeding pond where male densities are low and therefore mate choice could play a more prominently role; and during scramble competition within the pond where male densities and competition are high. We hypothesize that:

1) Smaller males should be faster in grabbing a female, if larger males have an advantage in male–male competition. As male–male competition is supposedly stronger within ponds, pairs caught within ponds should therefore show stronger size assortment than pairs caught outside ponds.
2) Larger individuals arrive first at the breeding sites, and size matching of pairs is partly due to temporal migration patterns.
3) If the relative distance between cloacae affects the fertilization success of pairs during amplexus, we expect size-matched pairs to show a greater fertilization success.

## MATERIAL AND METHODS

### Study areas

The study was carried out at 2 areas in southern and central Germany. The first is located in the deciduous beech forest surrounding the village Fabrikschleichach, Lower Franconia (49.924 N, 10.555 E; hereafter FS). This area contains a network of 140 ponds, where *R. temporaria* annually uses between 35 and 40 ponds for reproduction. In 2010, and in 2013 to 2016, we fenced 3–6 ponds, which have been continuously used for reproduction since 2005 (Grözinger et al. 2012), in order to catch pairs and single individuals outside the ponds. The fence consisted of plastic gauze (mesh size 2 mm, height approximately 60 cm) stretched between wooden poles. The ponds remained fenced for the entire reproductive period (1–2 weeks; 2010: 17–31 March; 2013: 02–17 April; 2014: 15–21 March; 2015: 14–31 March; 2016: 15 March–01 April). We installed buckets buried to the ground level along the exterior fence side (every 5 m), to collect arriving individuals. Fence and buckets were controlled twice a day (morning and evening), and all individuals (nonpaired males, *n* = 714; nonpaired females, *n* = 193) and pairs (*n* = 597) found were sexed and measured in situ. We measured snout-vent-length (SVL) using a caliper (in mm, to the closest 0.5 mm), and mass using a spring scale (1–100 g, 1 g increments). Additionally, the date and mating status (nonpaired or in amplexus) of arriving individuals was noted.

The second field site was located near Braunschweig, Lower Saxony, Germany. Here, fieldwork was carried out at the locality Kleiwiesen (52.328 N, 10.582 E; hereafter KW), which comprises a system of ponds surrounded by meadows and mixed deciduous beech forest, sustaining a large population of *R. temporaria*. According to our observations over a period of 10 years, almost the complete population breeds in a small shallow part of one pond, partly covered with dense reeds. Field observations were primarily carried out at night and began when the first pair was found and ended when there were no more pairs found (10–26 March 2012 and 08–16 April 2013). We caught all pairs (*n* = 174), nonpaired males (*n* = 412) and nonpaired females (*n* = 8) by hand from within the ponds and measured them on site for SVL and weight. Individuals were released only after completing measuring procedures to avoid recaptures.

### Size-assortative mating in the field

We tested if size-related mating patterns in *R. temporaria* are nonrandom and measured snout-vent-length SVL of nonpaired and paired individuals in different years and locations. Size data (SVL) of pairs were tested for their relationship with a Pearson correlation and the respective 95% confidence interval was calculated. In FS we found pairs of *R. temporaria* along the fence and within buckets. The latter theoretically could lead to biased results, i.e. larger males replacing smaller, already amplectant ones, especially in buckets where several pairs were trapped simultaneously. Therefore, we conducted separate analyses for pairs in and outside of buckets. For all statistical analyses, we used R software (Version 3.4.0., R Core Team 2017). The package ggplot2 was used for visualization (Wickham 2009). The mean SVL of paired versus nonpaired males and females was compared in each population with a Welsh 2 sample *t*-test and Cohen’s *d* was calculated as standardized effect size (R package effsize; Torchiano 2017). If differences were present, this would be a sign for nonrandom mating patterns, mate choice behavior and/or male–male competition. Furthermore, we calculated the intensity of sexual selection (ISS), defined as the standardized difference between the mean size of paired males and the mean size of all males in the population (Arnold and Wade 1984). This metric presents the shift of the mean value, caused by selection, in units of standard deviations for the specific phenotypic trait (Arnold and Wade 1984). The values of the male–female size ratio, defined as the SVL of the male divided by the SVL of the female, were compared with a Welsh 2 sample *t*-test to examine if size matching differed between locations. We compared the size matching ratios of our natural populations to the values we achieved with artificial pairing during the fertilization success experiment, to make sure the latter represent ratios found in nature.

### Temporal migration pattern

Temporal or spatial migration patterns of differently sized animals can lead to incidental assortment at the breeding site (Bearhop et al. 2005; Jiang et al. 2013). It is known from some explosive breeding anurans that larger males arrive first at the breeding site (Howard and Kluge 1985; Elmberg 1990). For the FS population, we collected data on day of appearance at the fence and tested if body size was decreasing with migration time, which could lead to an incidental size assortment during migration (total individuals: *n* = 2098). We fitted a linear mixed model (LMM) on body size with day of appearance and sex as fixed factors and year as random factor. To fit the model we used the lmer function in the R package lme4 (Bates et al. 2015) with restricted maximum likelihood and calculated the marginal and conditional coefficients of determination (R^2^) with the R package MuMIn (Bartoń 2016). The influence of fixed effects was tested with a Wald Chi^2^ test and that of the random effect with a restricted likelihood ratio test (RLRsim package; Scheipl et al. 2008).

### Mating speed experiment

**Figure 5:**
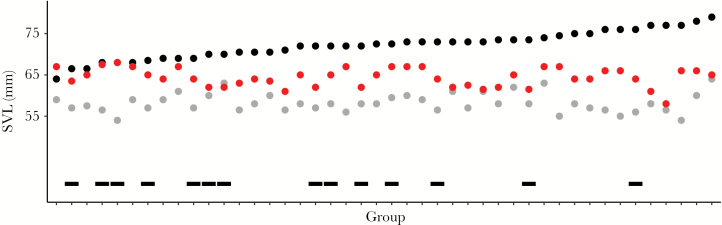
Mate speed experiment. Small males (gray), large males (black), and females (red) per trial. Black bars indicate the large male grabbing the female first. (*n* = 44). The groups are sorted by increasing large male SVL.

To test if male body size has an effect on time until mating in *R. temporaria*, we carried out a mating speed experiment in KW. Differently sized males in breeding condition; 1 large male (64–79 mm SVL) and 1 small male (54–64 mm SVL); were confronted with gravid females (58–68 mm SVL, in-between the SVL values of the 2 males, Figure 5). We then recorded occurrence of amplexus and the size of the successful male. This experiment was short-term, amplexus typically occurring within minutes and rarely after periods >1 h. Spawning did not occur in any of the trials. This experiment aimed to see which male (small vs. large) is faster in grabbing a medium sized female. The 3 test subjects were placed together in a water-filled container (diameter ca. 30 cm; water depth ca. 15 cm), and as soon as 1 male was observed in amplexus with a female it was recorded whether it was the smaller or the larger male. Each specimen (88 males, 44 females) was used for a single trial only (*n* = 44). The data were analyzed with a binomial test, where “small male grabs the female first” was defined as success. Additionally, we calculated a logistic regression model with binomial distributed response variable (success = small male first, failure = large male first) to find the variables that explain the observed pattern best. R^2^ was calculated with the MuMIn package (Bartoń 2016). We used the Zelig package (Choirat et al. 2016) to simulate the probability of the small male winning in dependency of the large males’ body size by using the logistic regression model. Therefore, we set mean size of females and mean size of small males as fixed variables and run the simulation over the range of the large males SVL with 1000 simulations.

### Fertilization success experiment

This experiment was designed to test whether assortative mating might confer a direct selective advantage by avoiding low fertilization rates that are known to occur with large size differences in anuran pairs (Davies and Halliday 1977; Robertson 1990). Therefore, we collected amplectant pairs in the KW area between 21 March 2015 and 1 May, 2015 (*n* = 45). Pairs were disengaged and transferred to the laboratory in buckets filled with water. In the lab, new pairs were placed separately in plastic tanks (dimensions 40 cm length, 22 cm width, 13 cm height) with 5–10 cm water and kept in a ventilated basement experiencing natural daily fluctuations in air pressure and temperature (tank water temperature mean ± SD = 12.0 ± 1.8°C, range = 6.5–14°C; measured by a iButton® Thermochron at 1 h intervals). The SVL of males and females was measured with a caliper to the nearest 1 mm, and pairs were arranged to achieve a broad range of size ratio values. We counted the total number of eggs produced within 48 h after spawning by placing the clutch in a light yellow plastic container to assure a high contrast between box and eggs. Eggs were carefully distributed across the bottom of the containers with little water and later slightly flattened with the aid of a transparent acrylic sheet. We took photographs and processed them with the spot detection function in Icy software (de Chaumont et al. 2012). After 7 days, another picture was taken and the number of undeveloped eggs was counted on screen to guarantee a precise discrimination of eggs and early larvae (Gosner stages 17–20, Gosner 1960). The task of automatically recognizing and counting the larvae in the 7 days clutch pictures was complicated, as they move and adopt many different shapes hence, instead of counting the larvae we counted the number of remaining (undeveloped) eggs, a much simpler image recognition task, and used the initial egg count as a reference for the calculations. We removed one completely unfertilized clutch from the dataset (total *n* = 44). The fertilization success was defined by the ratio of developed larvae to the number of deposited eggs (expressed as percent). For statistical analysis, we used a logistic regression model with binomial distribution of the response variable (success = number of embryos, fail = number of unfertilized eggs) and size-ratio of pairs as explanatory variable. A second approach was looking at male SVL as explanatory variable for fertilization success (logistic regression with binomial distribution). The SVL of male anurans could influence the fertilization success because bigger males produce a higher number of spermatozoa (Smith-Gill and Berven 1980; Edwards et al. 2004).

## RESULTS

### Size-assortative mating in the field

**Figure 1:**
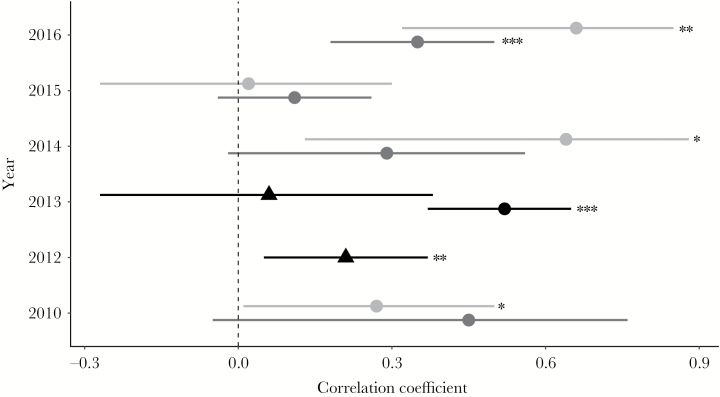
Correlation coefficient with respective 95% confidence interval of size assortment (snout-vent-length) of amplectant pairs of *R. temporaria* in the localities Fabrikschleichach (circle) and Kleiwiesen (triangle). Correlation coefficients are given for each year and are separated by pairs (black), pairs found inside of buckets (dark gray) and outside buckets at the fence (light gray). The black dotted line represents zero correlation. Significant correlations are marked with * *P* < 0.05, ** *P* < 0.01 and *** *P* < 0.001.

We detected positive size-assortative mating in both locations in almost all years, except in FS 2015 and KW 2013. The Pearson correlation coefficient (*r*) and the corresponding 95% confidence interval (CI) per year are given in Figure 1. The degree of assortment differed between pairs which were formed within buckets (pair in) and those that formed outside of buckets (pair out) but none of the groups showed consistently higher levels of assortment. In general, the CI increased with decreasing sample size and OSR had no influence on degree of assortment (Table 1). Detailed values per day can be found in the supplementary material (Supplementary Table S1).

**Table 1:** Summary of body sizes of *R. temporaria* pairs (Males and Females) found in 2 populations over several years, with effect size Cohen′s d and intensity of sexual selection

year	site	status	*n*	OSR	SVL M	*d* M	*d* CI 95%	ISS M	SVL F	*d* F	*d* CI 95%	ISS F
2010	FS	pair_in	17	1.2	69.18	0.64	0.03–1.24	0.87	71.14	0.34	−0.35–1.02	0.04
		pair_out	55		67.25	0.15	−0.28–0.75	−0.04	71.54	0.28	−0.25–0.81	0.19
2013		pair	107	1.5	70.8	−0.12	−0.37–0.13	−0.18	74.74	0.51	0.16–0.85	0.44
2014		pair_in	39	1.3	70.65	0.01	−0.42–0.42	0.11	75.51	0.29	−0.19–0.77	0.3
		pair_out	13		68.85	−0.34	−0.96–0.28	−0.76	75.46	0.3	−0.37–0.97	0.28
2015		pair_in	176	2.1	69.96	−0.05	−0.23–0.14	−0.07	74.45	0.32	−0.1–0.74	0.17
		pair_out	48		70.08	−0.03	−0.33–0.28	−0.02	73.27	0.15	−0.34–0.64	−0.29
2016		pair_in	121	1.5	64.17	−0.13	−0.36–0.11	−0.21	67.41	0.09	−0.21–0.39	0.1
		pair_out	21		65.43	0.08	−0.38–0.53	0.74	66.76	0.01	−0.5–0.49	−0.15
2012	KW	pair	137	2.5	71.19	0.48	0.26–0.69	0.68	66.73	0.49	−0.23–1.21	0.07
2013		pair	37	3.3	73.96	0.53	0.13–0.93	0.81	71.65	NA	NA	NA

Sites: FS = Fabrikschleichach, KW = Kleiwiesen; Status: pair in = inside of buckets, pair out = outside of buckets; OSR: operational sex ration (*n* males/*n* females per year); *d* = Cohen’s *d*; *d* CI 95%: corresponding 95% confidence interval of *d*; ISS: intensity of sexual selection.

**Figure 2:**
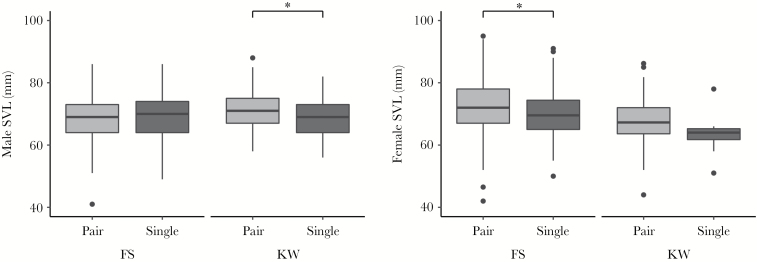
Differences of SVL between paired and nonpaired *R. temporaria* males (left) and females (right) at both study sites. Although no size differences were detected in Fabrikschleichach (FS) between paired (*n* = 597) and nonpaired males (*n* = 709), in Kleiwiesen (KW) paired males (*n* = 174) were significantly larger than nonpaired males (*n* = 285; *P* < 0.001, *d* = 0.43). Paired females (*n* = 597) in FS were significantly larger than nonpaired females (*n* = 193; *P* < 0.001, *d* = 0.35). In KW size difference was not significant; paired females (*n* = 174) did not differ from nonpaired ones (*n* = 8; *P =* 0.17, *d* = 0.63). The boxplots show median (dark line), 25–75% quartile (box), nonoutlier range (vertical line) and outliers (black dots). An asterisk depicts significant differences (*P < 0.05).

The SVL of amplectant males did not differ from that of nonpaired males in FS, where pairs were intercepted while migrating to the ponds (Welch 2 sample *t*-test, *t* = −1.39, *P* = 0.1661, *d* = −0.08) and in most years we observed only negligible effect sizes, i.e. variance of body size between the groups is not different from the variance within the group (Table 1). The intensity of sexual selection (ISS) was mostly small and negative in FS, showing that paired males were slightly smaller than nonpaired males (Figure 2, Table 1). Paired females in FS were significantly larger than nonpaired ones (Welsh 2 sample *t*-test, *t* = 4.20, *P* < 0.001, *d* = 0.35) and effect sizes were always positive with small to medium effect, i.e. the variance of body size between groups is higher than within the group and can be explained by the “effect” pairing. The ISS was mostly positive (Table 1).

**Figure 3:**
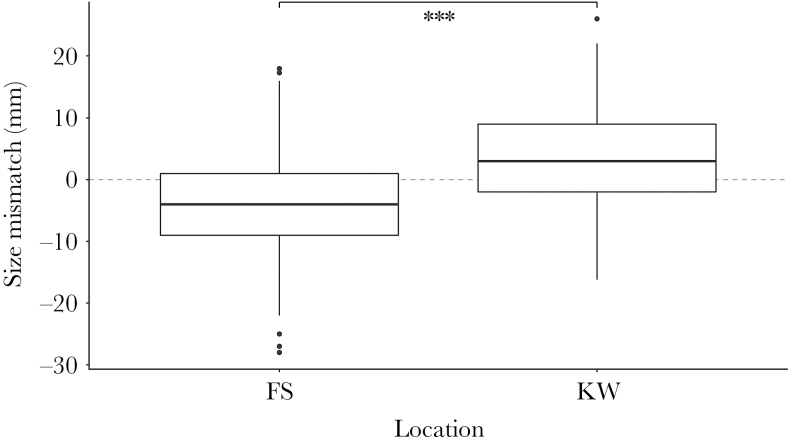
Size ratio of amplectant pairs of *R. temporaria* in Fabrikschleichach (males were smaller than females, *n* = 597) and Kleiwiesen (males were bigger than females, *n* = 174). The boxplots show median (dark line), 25–75% quartile (box), nonoutlier range (vertical line) and outliers (black dots). The dashed line depicts equal size of male and female. Between locations the difference of size ratio in pairs was significant and is depicted by 3 asterisks (Welsh 2 sample *t*-test, *t* = −10.54, *P* < 0.001, *d* = −0.99).

In KW, where pairs and single individuals were sampled in the breeding pond, and thus with potentially higher levels of scramble competition than in FS, the amplectant males were significantly larger than nonpaired ones (Welch 2 sample *t*-test, *t* = 4.38, *P* < 0.001, *d* = 0.43, Figure 2). When comparing paired males with all males within a population, the ISS was higher in KW than in FS (Table 1). Paired females were larger in KW (Figure 2). However, this difference was not significant (Welsh 2 sample *t*-test, *t* = 1.51, *P* = 0.1724, *d* = 0.63), probably due to small sample size of nonpaired females (*n* = 8). The male to female size ratio (measured as male SVL divided by female SVL) differed significantly between the locations (Welsh 2 sample *t*-test, *t* = −10.54, *P* < 0.001, *d* = −0.99). In FS, the mean pair size ratio was less than 1, (mean ± SD; 0.95 ± 0.10), i.e. females were larger than males (mean ± SD; SVL males: 68.6 ± 6.2 mm; SVL females: 72.4 ± 7.5 mm); although males were larger than females in KW (mean ± SD; 1.06 ± 0.12; SVL males: 71.2 ± 6.0 mm; SVL females: 67.8 ± 6.5 mm) (Figure 3).

### Temporal migration pattern

**Figure 4:**
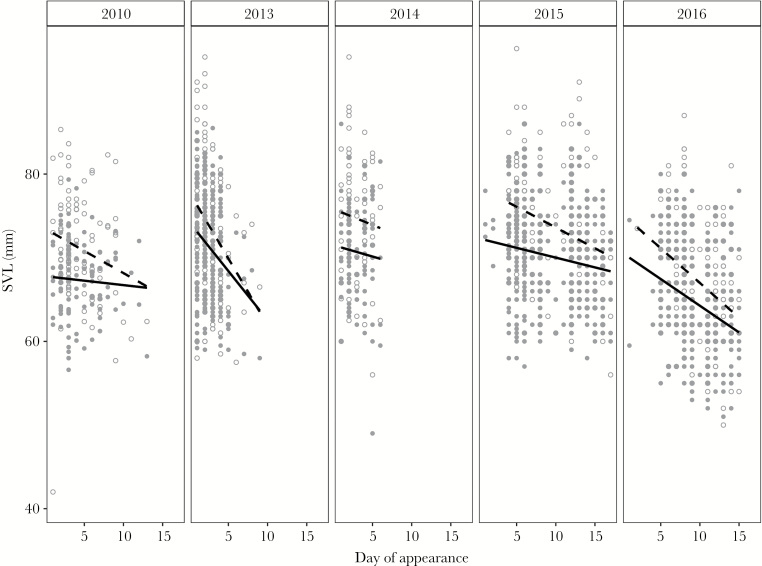
Relationship of appearance day at the fence (FS) and SVL in *R. temporaria* (open circles = females, filled circles = males). The plots show individual data per year and the respective linear regression line (dashed line = females, solid line = males).

At fenced ponds in FS, we found a consistent pattern of large specimens arriving earlier at the pond for both sexes, at almost all years (Figure 4). The LMM showed that body size was decreasing with ongoing time of migration within the year (Wald Chi^2^ test, χ^2^ = 108.26, df = 1, *P* < 0.001) for both sexes and that males were smaller than females (Wald Chi^2^ test, χ^2^ = 136.88, df = 1, *P* < 0.001). The fixed effects day and sex explained 13% of variance in our model (marginal R^2^). The year had an influence on frogs’ sizes (restricted likelihood ratio test, RLRT = 287.77, *P* < 0.001); when including year as random effect, the model explained 25% of variation in size differences (conditional R^2^, Supplementary Table S2).

### Mating speed experiment

**Figure 6:**
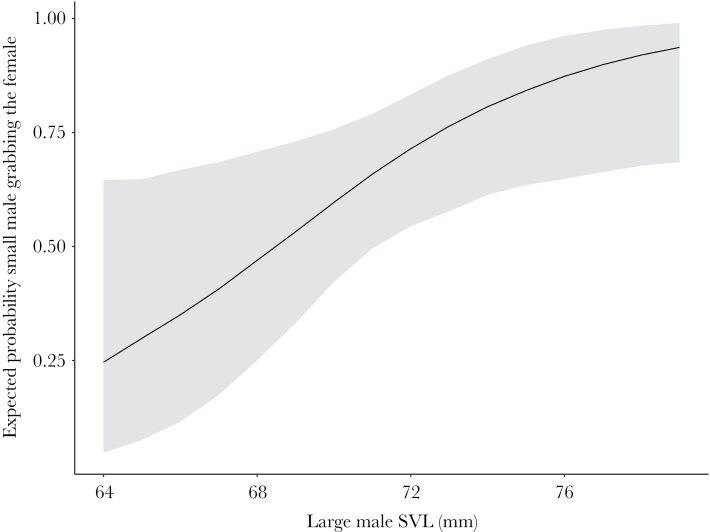
Probability of the small male grabbing the female first. Simulation was done with average female SVL (64.4 mm), average small male SVL (58.2 mm) and the range of large male SVL (64–79 mm). Given is the median probability of the small male grabbing the female first (black) and the 95% confidence interval (gray). The probability of the small male grabbing the female first is increasing with the increase of the large male’s body size.

Small males were 36% more successful (faster) in grabbing females than respective larger males. From 44 trials, smaller ones won in 30 of them (*n* = 44, 2-tailed Binomial test, *P* = 0.02, Figure 5). The logistic regression model for this experiment (*n* = 44) suggested that large male SVL has an influence on the winning probability of the small male (Z = 2.1, *P<* 0.05, Supplementary Table S3). In our simulation, we could see that the probability of the small male grabbing the female first increases as the SVL of the relatively larger male in an experiment increased (Figure 6). This indicates that the larger males got slower. Additionally, the size difference between the relatively larger male and the female seems to play a role. We observed a smaller size difference of large male to female when the large male grabbed her first (mean ± SD; 6.7 ± 3.9 mm) compared to when the small male grabbed her first (mean ± SD; 8.4 ± 4.5 mm). However, this difference was not significant (Welsh *t*-test, *t* = 1.26, *P* = 0.219, *d* = 0.39).

### Fertilization success experiment

**Figure 7:**
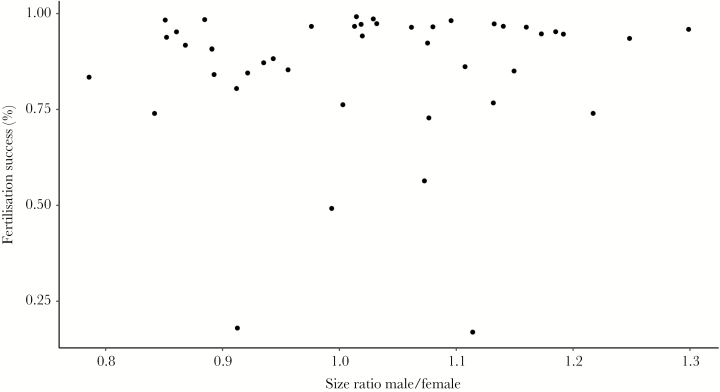
Relationship between size ratio (male/female SVL) and fertilization success in pairs of *R. temporaria* (*n* = 44). We could not detect any relationship between size ratio of pairs and fertilization success.

A total of 44 pairs mated and deposited eggs successfully in the laboratory. The male/female SVL ratio of breeders ranged between 0.78 and 1.30 (mean ± SD; 1.02 ± 0.13). The number of deposited eggs per female (mean ± SD, range; 1259 ± 384, 653–2213 eggs) was positively correlated with female size (mean ± SD, range; 65.5 ± 5.6, 55.3–80.3 mm; Pearson *r* = 0.90, 95% CI = 0.83–0.95, *P* < 0.001), where female size is accounting for approximately 80% of the variation in number of deposited eggs (linear regression, R^2^ = 0.81, F_(1,42)_ = 168.2, *P* < 0.001). The average fertilization success was relatively high but showed a wide range (mean ± SD, range; 85.6 ± 18.6%, 16.9–99.2% fertilization success, Figure 7). The logistic regression analyses showed no influence of size-ratio values or male SVL on fertilization success. The fit of these models was very poor and explained almost none of the variation in the dataset. Therefore, with our experimental approach, we could not detect an influence of size ratio on fertilization success.

## DISCUSSION

### Indications for a complex, multicausal size-assortative mating pattern

As expected from other studies (Berven 1981; Arak 1983; Gibbons and McCarthy 1986; Vojar et al. 2015), we detected positive size-assortative mating in both *R. temporaria* populations, and we observed body size differences between paired and nonpaired individuals. Such a size-assortative pattern is typically interpreted as a consequence of male mate choice. We observed a migration pattern, where larger individuals arrive first at the breeding pond, which supports size assortment by temporal covariation. However, our experiment indicated that mating tactics differ between small and large-sized males. The small males appear to be faster in grabbing a female than larger males. This pattern suggests that the explanation of size-assortative mating in these frogs is not as straightforward as it might seem at the first glance. In the following, we discuss the evidence for 3 main factors that might influence size-assortative mating in this species: temporal migration pattern, competitive male–male displacement and different mating tactics, and increased fertilization success of size-matched pairs.

### Size assortment during migration

In FS, we fenced the pond before migration started, and single frogs and pairs were intercepted at the fence. The pairs forming terrestrially, outside of the breeding aggregation, had to face a lower male density, resulting in a lower operational sex ratio, and therefore less competition between males (Höglund 1989; Byrne and Roberts 2004). Paired and nonpaired FS males did not differ in body size with a tendency of smaller males being paired, which supports the theory of less competition and the absence of large male advantage. The chances for smaller males to gain access to a large high quality female should be higher at low male densities (Arak 1988). Therefore, we expected a lower strength of size assortment. Still, we observed a positive size assortment of pairs, which could be due to temporal covariation that we observed at the fence, where larger individuals (males and females) arrived first at the breeding site. So far, this has been reported for males (Howard and Kluge 1985; Loman and Madsen 1986; Elmberg 1990) and rarely for females (Lodé et al. 2005), but the reasons behind this arrival pattern are not fully clarified (Wells 2007). The pattern could be due to physiological reasons, e.g. higher energy constrains of small individuals (Ryser 1989) or desiccation risk (Thorson 1955), which limits migration time and distances. Also, simple mechanistic reasons could lead to an earlier arrival of large individuals, since they are faster and can cover larger distances (Zug 1978). However, migration distances are highly variable (Pasanen and Sorjonen 1994; Kovar et al. 2009). Former experience of finding breeding ponds by larger, and thus older, individuals knowing the available sites better could be an important factor (Reading 2001). In addition, timing of arrival at the breeding site can influence survival of adults and eggs/larvae, either through unfavorable weather conditions like freezing or heat waves (Pasanen and Karhapää 1997; Håkansson and Loman 2004), or through predation (Heusser 1970; Lodé et al. 2004). Larger animals are less prone to freezing or desiccation, due to their surface/volume ratio; and they are too big for some predators. Furthermore, reproductive success can be influenced by arrival time, e.g. multiple mating in males can cause depleted sperm storages, energy reserves, or decreased mating motivation (Smith 1976; Gibbons and McCarthy 1986; Elmberg 1991; Hettyey et al. 2009a), which could therefore affect females that arrive late in the breeding season. Despite temporal covariation, size assortment during migration could be due to different mating tactics shown by small and large males, which are mostly density dependent (Arak 1988; Lodé et al. 2004). It can be expected that all males show a preference for large females to maximize their reproductive output, and that this preference is highest when there is less competition and therefore less costs (Fawcett and Johnstone 2003). This hypothesis is supported by our observation of paired females being larger than nonpaired females. Arriving early and grabbing a (high quality) female could have energetic benefits for small and large males, because the rate of male replacement is considered to be very low in most anurans (<5%, Wells 2007). Pairs of *R. temporaria* have been seen in amplexus for several hours or days without spawning (Geisselmann et al. 1971; Elmberg 1990; and own observations), which is considered to be a strategy of mate guarding (Savage 1961; Wells 1977; Arak 1983). However, the strategy of a prolonged amplexus could likewise favor female mate choice (Krupa 1995). It was shown that females are able to retain eggs if in amplexus with an unfavorable male and to prolong the pre-spawning period (Reyer et al. 1999; Hettyey et al. 2009b). Extended egg retainment (e.g. over several days) would provide females the possibility to test the endurance of the amplecting male, eventually leading to displacement of less perseverant males by others (Hettyey et al. 2009b). A *R. temporaria* male tactic to reduce the period during which they are exposed to possible male–male displacement fights might be to induce spawning by the application of pheromone proteins through the skin-abrasions on the female belly generated during amplexus (Willaert et al. 2013). However, more research is needed to test whether small males might produce these amplexin peptides more readily or in higher quantities to counteract potential female choice.

We believe that the size assortment in FS is primarily a result of temporal covariation during migration, subsequently modulated by mate choice behavior and low male–male competition.

### Size assortment during scramble competition

It has been previously shown that higher male densities in *R. temporaria* lead to a stronger size assortment (Vojar et al. 2015) and large-male advantage (Arak 1983; Elmberg 1991). In KW, the pairs were caught within the pond breeding aggregation where male densities are higher than in the terrestrial environment. Here, we observed paired males to be larger than nonpaired males, which could be a consequence of large-male advantage during scramble competition (Wells 1977; Höglund 1989; Byrne and Roberts 2004). For larger males it could pay off to fight for a large female, in order to maximize the potential number of eggs to fertilize, because large females have higher fecundity (our results; Howard and Kluge 1985; Ryser 1989; Elmberg 1991; Lardner and Loman 2003). Costs for maintaining a high quality female could thus be comparatively high for small males, as losing such a female to a larger male during scramble competition seems likely. Thus, the most successful tactics available for small males should be a prudent choice of smaller females (Härdling and Kokko 2005); or the unselective tactic of immediately grabbing any female. We saw in our mating speed experiments that on average, smaller males were faster in grabbing a female, which could be a consequence of unselective behavior of small males (Wells 1977), of the prudent choice of at least some small-sized males (Härdling and Kokko 2005), or simply of small males being more agile and swift in grabbing a female than their larger competitors. However, it is also possible that the females in the experiments were too small to trigger an amplexus behavior in the larger male (Kroupa 1995), as they were always—at least a bit—smaller than the larger male.

To conclude, male–male displacement in *R. temporaria* certainly occurs, but male–male displacement fights are not the immediate consequence of most encounters of single males with pairs, probably also due to the fact that single males might employ different mating tactics, like prudent or indiscriminate mate choice.

### Size-assortative mating and fertilization success

A positive size assortment could also arise from an active choice of similarly sized mates. Such a behavior may be adaptive if fertilization of eggs was compromised in size-mismatched pairs. In anurans, the influence of male/female size ratios on fertilization success is highly variable (Wogel et al. 2005). Experimental evidence in *R. temporaria* is mixed. Gibbons and McCarthy (1986) found a positive influence on the fertilization success when males have been larger, whereas Elmberg (1991) found the fertilization success being all or none and independent from male/female size ratio. Likewise, we could not detect a relationship between the size ratio of pairs and the percentage of fertilized eggs. In our experiment, the size ratio of pairs was comparable to the ones found in our natural populations and in former experimental studies (Gibbons and McCarthy 1986), and fertilization rates were comparable with former studies in nature (Gibbons and McCarthy 1986; Vieites et al. 2004). However, fertilization success is influenced by many different factors, which might confound such experimental results. This includes temperature, acidity (Beattie 1980; Freda 1986), number of former mating by the male (Gibbons and McCarthy 1986; Hettyey et al. 2009a) or the timely synchronization of gamete output/ejection, as well as sperm quality, sperm competition and genetic compatibility (Dziminski et al. 2009; Sherman et al. 2010; Álvarez et al. 2014).

Also, multiple paternities are a common phenomenon in lek-breeding anurans (Roberts et al. 1999; Lodé and Lesbarrères 2004; Merilä and Knopp 2009). In *R. temporaria,* multiple paternities can be caused by “clutch piracy”, where a satellite male grasps a clutch and fertilizes the eggs in the center of the clutch (Vieites et al. 2004; see Supplementary S4 for an example video of a breeding aggregation in FS, where a couple of nonpaired males enter a freshly laid clutch), but theoretically might also be caused by “stray sperm” (Laurila and Seppä 1998). In our experiment, stray sperm could have increased fertilization success in the limited amount of water, however, similar fertilization rates have been observed in nature (Gibbons and McCarthy 1986; Vieites et al. 2004).

## CONCLUSION

The complex mating system of *R. temporaria* is embedded in a multicausal framework. We believe that the temporal migration pattern plays an important role in the formation of size assortment during the migration period. If large males arrive earlier at the pond and gather in the shallow parts where spawning takes place, it would be beneficial for later arriving small males to stay at the pond edges and wait for the arriving females. As shown before, the large males have an advantage in scramble competition, are probably more successful in takeover attempts and could better hold on to a female. But, if a small male can grab a female before she is entering the breeding aggregation, the chances to stay with her until spawning occurs are good. Under higher levels of male competition, prudent or indiscriminate mate choice could be a successful mating tactic for smaller males. Therefore, size assortment is modulated by temporal covariation, male–male competition, male mate choice behavior, and seems to have no effect on fertilization success.

## SUPPLEMENTARY MATERIAL

Supplementary material can be found at https://academic.oup.com/beheco/

Supplementary Tables

Supplementary Video S4

## FUNDING

The field work in Fabrikschleichach was partly supported by an innovation fond of Museum für Naturkunde Berlin. C.D. was funded by a PhD scholarship from Elsa-Neumann Foundation of the Federal State of Berlin. The fee for the open access option was funded by the Museum für Naturkunde Berlin.

## Author’s Contribution

Designed study: C.D., H.F., M.V., A.R., M.O.R.; collected field and experimental data: C.D., A.R., O.S., H.F., S.D., M.V., M.O.R.; analyzed data: C.D., A.R., O.S.; drafted manuscript: C.D., H.F., M.V., A.R., M.O.R. All authors read, commented on, and approved the final version of the manuscript.

## Conflict of Interest

We have no competing interest.

## Ethical Statement

Research and handling permissions, according to German nature protection laws, were given for fieldwork in Lower Franconia by Regierung Unterfranken. Fieldwork in Lower Saxony was carried out with permits of Stadt Braunschweig and Landkreis Helmstedt, and experiments approved by the ethics committee of Lower Saxony.

## Data accessibility

Analyses reported in this article can be reproduced using the data provided by Dittrich et al. (2017).
