# Supplementary material for: Temporal migration patterns and mating tactics influence size-assortative mating in Rana temporaria
Source: Behav Ecol. 2018 Jan 10;29(2):418–28. doi: 10.1093/beheco/arx188 (PMC5873255; doi:10.1093/beheco/arx188)
Supplement: Supplementary Tables [file arx188_suppl_supplementary_tables.doc]

**Supplement S1-S3**

**Table S1** Summary of different variables per day for each year and location: number of males (N_M_all) and females (N_F_all), SVL of all males (SVL_M_all) and females (SVL_F_all), number of pairs per day (N_pairs), SVL of males and females in the pairs (SVL_M_pair and SVL_F_pair), operational sex ration (OSR), correlation coefficient after Pearson (cor_coef) with respective P-value (P) and lower and upper confidence interval (ci_low, ci_up). Data are separated by location: FS = Fabrikschleichach; KW = Kleiwiesen

| **day** | **year** | **location** | **N_M_all** | **SVL_M_all** | **N_F_all** | **SVL_F_all** | **N_pairs** | **SVL_M_pair** | **SVL_F_pair** | **OSR** | **cor_coef** | **P** | **ci_low** | **ci_up** |
| --- | --- | --- | --- | --- | --- | --- | --- | --- | --- | --- | --- | --- | --- | --- |
| 1 | 2010 | FS | 8 | 67.19 | 3 | 76.42 | 2 | NA | NA | 2.67 | NA | NA | NA | NA |
| 2 | 2010 | FS | 19 | 67.93 | 15 | 73.40 | 14 | 68.76 | 73.73 | 1.27 | 0.41 | 0.15 | -0.15 | 0.77 |
| 3 | 2010 | FS | 32 | 67.24 | 26 | 70.60 | 20 | 67.24 | 70.87 | 1.23 | 0.28 | 0.23 | -0.18 | 0.64 |
| 4 | 2010 | FS | 8 | 67.55 | 8 | 73.74 | 6 | 68.10 | 73.09 | 1.00 | -0.03 | 0.96 | -0.82 | 0.80 |
| 5 | 2010 | FS | 8 | 67.94 | 9 | 69.91 | 7 | 68.48 | 70.58 | 0.89 | 0.60 | 0.16 | -0.28 | 0.93 |
| 6 | 2010 | FS | 10 | 67.24 | 10 | 69.95 | 9 | 68.02 | 70.09 | 1.00 | 0.03 | 0.94 | -0.65 | 0.68 |
| 7 | 2010 | FS | 9 | 65.52 | 7 | 67.36 | 5 | 64.42 | 67.77 | 1.29 | -0.02 | 0.98 | -0.89 | 0.88 |
| 8 | 2010 | FS | 4 | 67.65 | 3 | 76.60 | 3 | 67.01 | 76.60 | 1.33 | NA | NA | NA | NA |
| 9 | 2010 | FS | 5 | 69.48 | 6 | 70.90 | 4 | 70.90 | 71.23 | 0.83 | -0.06 | 0.94 | -0.97 | 0.96 |
| 11 | 2010 | FS | 1 | 68.20 | 1 | 60.30 | 1 | 68.20 | 60.30 | 1.00 | NA | NA | NA | NA |
| 12 | 2010 | FS | 2 | 68.20 | 0 | NA | 0 | NA | NA | NA | NA | NA | NA | NA |
| 13 | 2010 | FS | 1 | 58.22 | 1 | 62.38 | 1 | 58.22 | 62.38 | 1.00 | NA | NA | NA | NA |
| 1 | 2013 | FS | 63 | 72.44 | 27 | 76.13 | NA | NA | NA | 2.33 | NA | NA | NA | NA |
| 2 | 2013 | FS | 82 | 72.16 | 65 | 75.35 | 53 | 71.65 | 76.51 | 1.26 | 0.47 | 0.00 | 0.22 | 0.65 |
| 3 | 2013 | FS | 53 | 71.44 | 26 | 72.12 | 18 | 70.44 | 72.36 | 2.04 | 0.45 | 0.06 | -0.02 | 0.76 |
| 4 | 2013 | FS | 34 | 70.46 | 25 | 70.72 | 17 | 70.82 | 72.26 | 1.36 | 0.15 | 0.57 | -0.36 | 0.59 |
| 5 | 2013 | FS | 9 | 63.06 | 5 | 69.00 | 4 | 62.63 | 70.75 | 1.80 | 0.97 | 0.03 | 0.19 | 1.00 |
| **day** | **year** | **location** | **N_M_all** | **SVL_M_all** | **N_F_all** | **SVL_F_all** | **n_pairs** | **SVL_M_pair** | **SVL_F_pair** | **OSR** | **cor_coef** | **P** | **ci_low** | **ci_up** |
| 6 | 2013 | FS | 1 | 73.00 | 2 | 66.25 | 1 | 73.00 | 75.00 | 0.50 | NA | NA | NA | NA |
| 7 | 2013 | FS | 3 | 66.17 | 3 | 67.83 | 1 | 72.50 | 73.00 | 1.00 | NA | NA | NA | NA |
| 8 | 2013 | FS | 1 | 68.50 | 1 | 74.00 | 1 | 68.50 | 74.00 | 1.00 | NA | NA | NA | NA |
| 9 | 2013 | FS | 1 | 58.00 | 1 | 66.50 | 1 | 58.00 | 66.50 | 1.00 | NA | NA | NA | NA |
| 1 | 2014 | FS | 12 | 71.12 | 10 | 71.73 | NA | NA | NA | 1.20 | NA | NA | NA | NA |
| 2 | 2014 | FS | 43 | 71.27 | 34 | 77.31 | 21 | 71.40 | 77.50 | 1.26 | 0.30 | 0.18 | -0.15 | 0.65 |
| 3 | 2014 | FS | 9 | 67.50 | 9 | 69.18 | 5 | 66.00 | 68.50 | 1.00 | 0.38 | 0.52 | -0.75 | 0.95 |
| 4 | 2014 | FS | 23 | 71.13 | 16 | 75.25 | 11 | 70.18 | 76.05 | 1.44 | 0.45 | 0.17 | -0.21 | 0.83 |
| 5 | 2014 | FS | 15 | 70.60 | 11 | 73.82 | 8 | 72.81 | 75.25 | 1.36 | -0.18 | 0.67 | -0.79 | 0.60 |
| 6 | 2014 | FS | 4 | 68.25 | 3 | 72.33 | 3 | 63.83 | 72.33 | 1.33 | NA | NA | NA | NA |
| 1 | 2015 | FS | 3 | 74.83 | 0 | NA | NA | NA | NA | 0.00 | NA | NA | NA | NA |
| 2 | 2015 | FS | 3 | 72.33 | 0 | NA | NA | NA | NA | 0.00 | NA | NA | NA | NA |
| 4 | 2015 | FS | 43 | 71.36 | 18 | 75.53 | 15 | 71.87 | 75.70 | 2.39 | 0.00 | 1.00 | -0.51 | 0.51 |
| 5 | 2015 | FS | 76 | 70.68 | 38 | 77.04 | 36 | 69.69 | 77.21 | 2.00 | 0.09 | 0.60 | -0.24 | 0.41 |
| 6 | 2015 | FS | 78 | 71.26 | 44 | 76.41 | 42 | 71.37 | 76.50 | 1.77 | 0.09 | 0.58 | -0.22 | 0.38 |
| 7 | 2015 | FS | 12 | 69.50 | 11 | 71.27 | 11 | 69.09 | 71.27 | 1.09 | 0.18 | 0.60 | -0.47 | 0.70 |
| 8 | 2015 | FS | 45 | 69.11 | 23 | 72.74 | 23 | 70.04 | 72.74 | 1.96 | 0.17 | 0.43 | -0.26 | 0.55 |
| 9 | 2015 | FS | 11 | 70.91 | 5 | 70.40 | 4 | 68.25 | 70.50 | 2.20 | 0.72 | 0.28 | -0.78 | 0.99 |
| 10 | 2015 | FS | 5 | 67.00 | 2 | 68.00 | 2 | 67.50 | 68.00 | 2.50 | NA | NA | NA | NA |
| 11 | 2015 | FS | 6 | 71.33 | 3 | 79.67 | 3 | 68.67 | 79.67 | 2.00 | NA | NA | NA | NA |
| 12 | 2015 | FS | 99 | 70.94 | 52 | 73.06 | 47 | 71.13 | 73.45 | 1.90 | -0.09 | 0.57 | -0.36 | 0.21 |
| 13 | 2015 | FS | 33 | 70.45 | 15 | 74.27 | 15 | 61.40 | 66.20 | 2.20 | 0.29 | 0.30 | -0.27 | 0.70 |
| 14 | 2015 | FS | 36 | 69.56 | 13 | 75.31 | 8 | 68.25 | 73.13 | 2.77 | -0.30 | 0.47 | -0.83 | 0.51 |
| 15 | 2015 | FS | 25 | 67.88 | 5 | 70.00 | 4 | 68.50 | 68.25 | 5.00 | -0.70 | 0.30 | -0.99 | 0.79 |
| 16 | 2015 | FS | 42 | 66.79 | 14 | 68.00 | 12 | 65.75 | 68.50 | 3.00 | -0.01 | 0.97 | -0.58 | 0.57 |
| 17 | 2015 | FS | 10 | 67.40 | 6 | 67.17 | 4 | 63.25 | 69.25 | 1.67 | 0.40 | 0.60 | -0.91 | 0.98 |
| 1 | 2016 | FS | 2 | 67.00 | 0 | NA | NA | NA | NA | NA | NA | NA | NA | NA |
| 2 | 2016 | FS | 0 | NA | 1 | 73.50 | NA | NA | NA | NA | NA | NA | NA | NA |
| 3 | 2016 | FS | 0 | NA | 0 | NA | NA | NA | NA | NA | NA | NA | NA | NA |
| **day** | **year** | **location** | **N_M_all** | **SVL_M_all** | **N_F_all** | **SVL_F_all** | **n_pairs** | **SVL_M_pair** | **SVL_F_pair** | **OSR** | **cor_coef** | **P** | **ci_low** | **ci_up** |
| 4 | 2016 | FS | 5 | 66.20 | 0 | NA | NA | NA | NA | NA | NA | NA | NA | NA |
| 5 | 2016 | FS | 26 | 67.04 | 12 | 70.25 | 11 | 65.09 | 70.73 | 2.17 | 0.55 | 0.08 | -0.08 | 0.86 |
| 6 | 2016 | FS | 31 | 68.06 | 14 | 71.50 | 12 | 67.25 | 72.25 | 2.21 | 0.76 | 0.00 | 0.32 | 0.93 |
| 7 | 2016 | FS | 19 | 67.58 | 13 | 67.62 | 8 | 68.00 | 69.00 | 1.46 | 0.33 | 0.43 | -0.49 | 0.84 |
| 8 | 2016 | FS | 72 | 66.19 | 43 | 70.81 | 27 | 66.52 | 70.33 | 1.67 | -0.03 | 0.88 | -0.41 | 0.35 |
| 9 | 2016 | FS | 40 | 63.50 | 24 | 66.00 | 15 | 64.13 | 66.07 | 1.67 | 0.47 | 0.08 | -0.06 | 0.79 |
| 10 | 2016 | FS | 2 | 59.00 | 4 | 67.25 | 2 | 59.00 | 68.00 | 0.50 | NA | NA | NA | NA |
| 11 | 2016 | FS | 38 | 62.42 | 35 | 63.69 | 23 | 61.43 | 62.96 | 1.09 | 0.33 | 0.13 | -0.10 | 0.65 |
| 12 | 2016 | FS | 21 | 62.62 | 23 | 65.48 | 14 | 63.36 | 65.57 | 0.91 | 0.18 | 0.54 | -0.39 | 0.65 |
| 13 | 2016 | FS | 22 | 61.95 | 22 | 66.14 | NA | NA | NA | 1.00 | NA | NA | NA | NA |
| 14 | 2016 | FS | 33 | 62.12 | 16 | 64.63 | 12 | 64.83 | 65.67 | 2.06 | 0.58 | 0.05 | 0.02 | 0.87 |
| 15 | 2016 | FS | 10 | 63.90 | 4 | 62.50 | 3 | 65.33 | 63.00 | 2.50 | NA | NA | NA | NA |
| 1 | 2012 | KW | 9 | 65.29 | 9 | 65.10 | NA | NA | NA | 1.00 | NA | NA | NA | NA |
| 2 | 2012 | KW | 2 | 67.25 | 2 | 63.30 | 2 | 67.25 | 63.30 | 1.00 | NA | NA | NA | NA |
| 3 | 2012 | KW | 17 | 66.39 | 17 | 62.82 | 17 | 66.39 | 62.82 | 1.00 | 0.07 | 0.79 | -0.42 | 0.53 |
| 5 | 2012 | KW | 80 | 67.91 | 23 | 65.96 | 18 | 70.83 | 66.06 | 3.48 | 0.38 | 0.12 | -0.11 | 0.72 |
| 6 | 2012 | KW | 41 | 68.78 | 23 | 65.87 | 22 | 70.59 | 65.95 | 1.78 | 0.02 | 0.92 | -0.40 | 0.44 |
| 7 | 2012 | KW | 53 | 69.64 | 25 | 68.28 | 24 | 69.38 | 68.38 | 2.12 | -0.22 | 0.31 | -0.57 | 0.21 |
| 9 | 2012 | KW | 40 | 72.65 | 30 | 68.80 | 30 | 74.03 | 68.80 | 1.33 | 0.26 | 0.17 | -0.11 | 0.57 |
| 10 | 2012 | KW | 51 | 70.78 | 15 | 67.73 | 15 | 72.40 | 67.73 | 3.40 | 0.04 | 0.87 | -0.48 | 0.54 |
| 12 | 2012 | KW | 35 | 63.31 | 0 | NA | 9 | 65.29 | 65.10 | 0.00 | 0.15 | 0.70 | -0.57 | 0.74 |
| 1 | 2013 | KW | 14 | 74.77 | 3 | 70.87 | NA | NA | NA | 4.67 | NA | NA | NA | NA |
| 2 | 2013 | KW | 6 | 74.02 | 6 | 73.42 | 6 | 74.02 | 73.42 | 1.00 | 0.46 | 0.36 | -0.56 | 0.93 |
| 3 | 2013 | KW | 9 | 72.82 | 9 | 69.09 | 9 | 72.82 | 69.09 | 1.00 | 0.34 | 0.37 | -0.42 | 0.82 |
| 4 | 2013 | KW | 58 | 71.51 | 7 | 72.86 | 7 | 71.73 | 72.86 | 8.29 | 0.07 | 0.88 | -0.72 | 0.78 |
| 5 | 2013 | KW | 5 | 77.10 | 5 | 74.64 | 5 | 77.10 | 74.64 | 1.00 | -0.68 | 0.21 | -0.98 | 0.51 |
| 6 | 2013 | KW | 1 | 74.10 | 1 | 70.70 | 1 | 74.10 | 70.70 | 1.00 | NA | NA | NA | NA |
| 7 | 2013 | KW | 4 | 69.00 | 4 | 70.38 | 4 | 69.00 | 70.38 | 1.00 | 0.34 | 0.66 | -0.92 | 0.98 |

**Table S2. Summary of the linear mixed model output for migration data in Fabrikschleichach, with estimates and standard deviation of fixed effects (day, sex), random effect (year) and model validation parameters.**

|  | Size (SVL) |
| --- | --- |
| day | -0.442 ± 0.043 |
| sex male | -3.218 ± 0.275 |
| constant | 74.648 ± 1.109 |
| n year | 5 |
| standard deviation | 2.364 |
| n | 2,098 |
| log likelihood | -6,771.854 |
| AIC | 13,553.710 |
| BIC | 13,581.950 |
| marginal R^2^ | 0.1314061 |
| conditional R^2^ | 0.2456540 |

**Table S3.** Summary of the mating speed behavior model (glm with binomial family). Given are estimates with standard error in brackets and model validation parameters.

=============================================

Dependent variable:

---------------------------

win

---------------------------------------------

small_male_SVL 0.228

(0.187)

large_male_SVL 0.259**

(0.124)

Female_SVL 0.205

(0.168)

Constant -44.211**

(21.914)

---------------------------------------------

Observations 44

Log Likelihood -24.153

Akaike Inf. Crit. 56.307

=============================================

Note: *p<0.1; **p<0.05; ***p<0.01
